# New Advances in Cardiovascular Drugs: In Memory of Professor Akira Endo

**DOI:** 10.3390/biomedicines14020487

**Published:** 2026-02-23

**Authors:** Alfredo Caturano

**Affiliations:** Department of Human Sciences and Promotion of the Quality of Life, San Raffaele Roma University, 00166 Rome, Italy; alfredo.caturano@uniroma5.it

## 1. Introduction: Honoring a Visionary Who Transformed Cardiovascular Medicine

In this Special Issue, we honor the memory of a giant within cardiovascular pharmacology, Professor Akira Endo, whose scientific vision profoundly transformed the prevention and treatment of cardiovascular disease. With his passing on 5 June 2024, cardiovascular medicine lost a scientist whose intellectual legacy is deeply woven into everyday clinical practice worldwide [1]. Professor Endo’s work fundamentally reshaped the management of atherosclerotic cardiovascular disease, moving the field from a largely reactive approach to one firmly grounded in mechanism-based prevention. His discovery marked a decisive turning point, demonstrating that targeted intervention on cholesterol metabolism could meaningfully alter the course of cardiovascular disease at both the individual and population level [2].

Beyond the introduction of a single class of drugs, Professor Endo’s contribution established a new scientific mindset that brought together biochemical insight, translational research, and long-term public health impacts. Statins became not only a therapeutic cornerstone but also a catalyst for decades of innovation, inspiring subsequent advances in lipid-lowering strategies and cardiovascular pharmacology. Millions of patients continue to benefit daily from this legacy, underscoring the profound and enduring societal relevance of his work. While Professor Endo is no longer with us, the principles he embodied, curiosity, perseverance, and a commitment to improving human health, remain central to the ongoing evolution of cardiovascular medicine and continue to guide future generations of researchers and clinicians.

## 2. Endo’s Legacy as the Scientific Backbone of the Special Issue

Cardiovascular medicine has witnessed numerous breakthroughs over the past few decades, yet only a limited number have achieved the same broad and sustained impact on global health as statins [1]. The scientific legacy of Akira Endo forms the conceptual backbone of modern cardiovascular pharmacology and exemplifies how deep mechanistic understanding can be translated into long-lasting clinical benefits. By identifying low-density lipoprotein cholesterol (LDL-C) as a modifiable driver of atherosclerotic disease, statins fundamentally redefined the goals and strategies of cardiovascular prevention [3].

This insight paved the way for the progressive evolution of cardiovascular risk management, extending beyond lipid lowering toward more refined approaches to risk stratification and individualized therapy [4]. Over time, statins became the foundation upon which combination regimens and novel pharmacological classes were developed, addressing the complex and multifactorial nature of cardiovascular disease [3]. Contemporary precision-based prevention strategies continue to follow the logical framework first established by Professor Endo’s work. In this respect, his contribution marked a decisive transition from the treatment of clinical manifestations to a mechanism-based, anticipatory model of care, a paradigm that continues to guide cardiovascular drug development and underpins the scientific rationale of this Special Issue [2].

## 3. Overview of Published Articles

Inspired by the scientific legacy of Professor Endo, cardiovascular pharmacology has continued to advance through innovations that expand and refine contemporary strategies for cardiovascular disease management. This Special Issue brings together contributions from established researchers in the field, each addressing a distinct aspect of cardiovascular drug development with potential relevance for future clinical practice.

Vitulano et al. (contribution 1) investigated the effects of a novel nutraceutical combination on lipid parameters in subjects with sub-optimal blood cholesterol levels and low–intermediate cardiovascular risk. In a cohort of 44 pharmacologically untreated individuals followed for six months, supplementation with a compound containing plant extracts and phytosterols resulted in a significant and sustained reduction in low-density lipoprotein cholesterol (LDL-C) and total cholesterol, while triglycerides remained unchanged and high-density lipoprotein cholesterol showed only transient variation. This study supports the potential role of targeted nutraceutical combinations as an adjunct or alternative strategy for cholesterol management, particularly in statin-intolerant patients or in those not eligible for pharmacological therapy.

Kapustová et al. (contribution 2) explored the pleiotropic effects of statins on the expression of genes associated with severe COVID-19 in both cancerous and non-cancerous cellular models. Using transcriptomic analyses of pancreatic cancer cells and adipose tissue-derived mesenchymal stem cells, the authors evaluated the impact of all currently available statins on genes linked to complicated COVID-19 outcomes. While statins did not directly alter the expression of key genes such as APOE and ACE2 or loci associated with respiratory failure, several statins modulated the expression of genes involved in inflammatory and immune pathways, including IL-6, IL-8, and NF-κB interaction partners. These findings highlight the complex, pathway-specific actions of statins and suggest that their net effect on COVID-19 may reflect a balance between pro- and anti-inflammatory mechanisms.

Otoda et al. (contribution 3) reviewed the emerging role of lysosomal stress as a key pathogenic mechanism in cardiovascular diseases, highlighting its involvement in chronic inflammation, lipid dysregulation, and oxidative injury. The authors discuss critical molecular pathways, including NLRP3 inflammasome activation, TFEB-mediated regulation of autophagy, ferroptosis, and the contribution of apolipoprotein M to lysosomal integrity, as well as the impact of impaired lysosomal acidification on cardiac function. The review also examines the therapeutic potential of several cardiovascular and adjunctive agents, such as statins, SGLT2 inhibitors, and bioactive compounds, in restoring lysosomal function and mitigating disease progression. Despite promising experimental evidence, the authors emphasize that major challenges still remain in biomarker development, targeted drug delivery, and clinical validation, underscoring the need for further translational research to integrate lysosome-focused strategies into cardiovascular therapy.

Salmen et al. (contribution 4) evaluated the real-world effectiveness of GLP-1 receptor agonists and SGLT-2 inhibitors in achieving combined glycemic control (HbA1c < 7%) and body weight reduction of at least 5% in patients with type 2 diabetes mellitus. In a retrospective cohort of 405 outpatients followed for up to 12 months, SGLT-2 inhibitors demonstrated superior performance compared with GLP-1 receptor agonists and metformin, showing the highest rates of combined target achievement over time. The study highlights the sustained efficacy of SGLT-2 inhibitors in routine clinical practice and underscores their growing role in the long-term management of type 2 diabetes, particularly when both metabolic control and weight reduction are therapeutic goals.

Bharaj et al. (contribution 5) reviewed contemporary and emerging therapeutic strategies for cardiovascular–kidney–metabolic (CKM) syndrome, a complex condition driven by the intersection of cardiovascular disease, chronic kidney disease, type 2 diabetes, and obesity. Framed in memory of Prof. Akira Endo, the review integrates evidence on established agents, such as SGLT2 inhibitors, GLP-1 receptor agonists, and nonsteroidal mineralocorticoid receptor antagonists, that have demonstrated benefits for cardiovascular, renal, and metabolic outcomes. The authors also highlight innovative approaches, including therapies targeting lipoprotein (a), inflammatory pathways, and hepatic steatosis and RNA-based modulation of PCSK9, alongside the emerging role of artificial intelligence in personalized risk assessment. This comprehensive overview underscores the shift toward integrated, mechanism-driven care for CKM syndrome, reflecting the evolving landscape of cardiometabolic therapeutics.

Jovanović (contribution 6) provided a comprehensive overview of cardioprotective signaling mechanisms, outlining both established concepts and future research directions in the field. Building on the original observation of ischemic preconditioning, the review describes how diverse stimuli, including metabolic stress, hypoxia, temperature changes, and pharmacological agents, activate convergent intracellular pathways that enhance cardiac resistance to injury. Key signaling networks such as the RISK and SAFE pathways, together with mediators including protein kinases, HIF-1α, microRNAs, and Connexin 43, are discussed regarding their roles in mitochondrial function, gene expression, and cellular homeostasis. The author highlights that despite substantial progress in delineating these molecular networks, the translation of cardioprotective signaling into effective and clinically applicable therapeutic strategies remains a major unresolved challenge.

Dass (contribution 7) reviewed the biological roles of pigment epithelium-derived factor (PEDF), focusing on its involvement in metabolic disease, diabetes, angiogenesis, and cardiovascular disease. The review summarizes evidence linking PEDF to key metabolic and signaling pathways, including insulin signaling, AMPK-α, PPAR-γ, Wnt/β-catenin, and oxidative stress, highlighting its pleiotropic effects across metabolic and cardiovascular systems. By integrating data from experimental and translational studies, the author discusses the dual physiological and pathological roles of PEDF and outlines future directions aimed at advancing toward clinical evaluation of this serpin as a potential therapeutic target.

Shahzad et al. (contribution 8) reviewed the effects of microgravity on cardiovascular structure and function, focusing on the cellular and molecular responses of different cardiac and vascular cell types. The authors summarize evidence showing that simulated microgravity alters cytoskeletal organization and endothelial permeability and migration and contributes to myocardial atrophy and endothelial dysfunction. They also discuss additional effects on coagulation, vascular tone, blood volume regulation, and cardiac geometry. This comprehensive review emphasizes that microgravity is a unique experimental condition for understanding cardiovascular adaptation and vulnerability, underscoring the need for mechanistic studies to inform both space medicine and terrestrial cardiovascular research.

## 4. Future Challenges and Unresolved Questions in Cardiovascular Pharmacology

Despite remarkable progress in cardiovascular pharmacology, the contributions included in this Special Issue collectively highlight that several critical challenges remain unresolved. One of the largest issues is the need to further refine risk stratification and therapy personalization. While lowering LDL-C remains a central pillar of prevention, it is becoming increasingly clear that cardiovascular risk is shaped by a complex interplay of metabolic, inflammatory, genetic, and environmental factors [5,6,7,8]. Translating this complexity into individualized treatment algorithms and identifying the right drug, or combination of drugs, for the right patient at the right time remains an open and pressing challenge. Another major unmet need is the integration of novel mechanisms into clinically actionable therapies. Advances in our understanding of pleiotropic drug effects, lysosomal stress, cardioprotective signaling pathways, and metabolic cross-talk have substantially expanded the mechanistic landscape of cardiovascular disease [9]. However, the gap between mechanistic insight and effective clinical translation persists. The development of reliable biomarkers, targeted drug delivery systems, and robust validation in large-scale clinical trials is essential if these promising strategies are to be moved from experimental settings into routine practice.

The growing burden of cardiovascular–kidney–metabolic syndrome further underscores the need to abandon siloed approaches to disease management [10]. Future therapies must be designed to address shared pathophysiological pathways across organ systems, rather than focusing on single disease entities. In this context, combination therapies, RNA-based drugs, and emerging digital tools, such as artificial intelligence for risk prediction and treatment optimization, offer significant promise but also raise new questions regarding long-term safety, accessibility, and cost-effectiveness [11,12]. Finally, emerging and unconventional research models, including extreme physiological conditions such as microgravity, challenge traditional views of cardiovascular adaptation and vulnerability. These models not only broaden our understanding of cardiovascular biology but also force the field to reconsider fundamental assumptions about tissue resilience, remodeling, and disease progression. Harnessing insights from such models may open unexpected avenues for therapeutic innovation, provided that mechanistic findings can be meaningfully translated to terrestrial clinical care.

In many ways, these challenges echo the scientific mindset exemplified by Prof. Akira Endo himself: the willingness to look beyond established paradigms, connect basic biology with clinical necessity, and pursue prevention as a long-term investment in public health. Addressing these unresolved questions will require the same combination of curiosity, rigor, and translational vision that defined Professor Endo’s work and ensure that his legacy continues to shape the future of cardiovascular medicine.

## Data Availability

No dataset was generated for the publication of this article.
